# Supplementary material for: Radical-Scavenging Activatable and Robust Polymeric Binder Based on Poly(acrylic acid) Cross-Linked with Tannic Acid for Silicon Anode of Lithium Storage System
Source: Nanomaterials (Basel). 2022 Sep 30;12(19):3437. doi: 10.3390/nano12193437 (PMC9565638; doi:10.3390/nano12193437)
Supplement: Supplementary file 1 [file nanomaterials-12-03437-s001.zip › nanomaterials-1900081-supplementary.pdf]

nanomaterials

Article

Radical-Scavenging Activatable and Robust Polymeric Binder Based on Poly(acrylic acid) Cross-linked with Tannic Acid for Silicon Anode of Lithium Storage System

Hui Gyeong Park [1,†], Mincheol Jung [2,3,†], Shinyoung Lee [4], Woo-Jin Song [2,3,4,*] and Jung-Soo Lee [1,*]

1 Department of Chemical Engineering, Graduate School of Chosun University, 309 Pilmun-daero, Dong-gu, Gwangju 61452, Korea
2 Department of Polymer Science and Engineering, Chungnam National University, 99 Daehak-ro, Yuseong-gu, Daejeon 34134, Korea
3 Department of Chemical Engineering and Applied Chemistry, Chungnam National University, 99 Daehak-ro, Yuseong-gu, Daejeon 34134, Korea
4 Department of Organic Materials Engineering, Chungnam National University, 99 Daehak-ro, Yuseong-gu, Daejeon 34134, Korea
* Correspondence: wjsong@cnu.ac.kr (W.-J.S.); jslee15@chosun.ac.kr (J.-S.L.); Tel.: +82-62-7163 (W.-J.S.); +82-62-608-5402 (J.-S.L.)
† These authors contributed equally to this study.

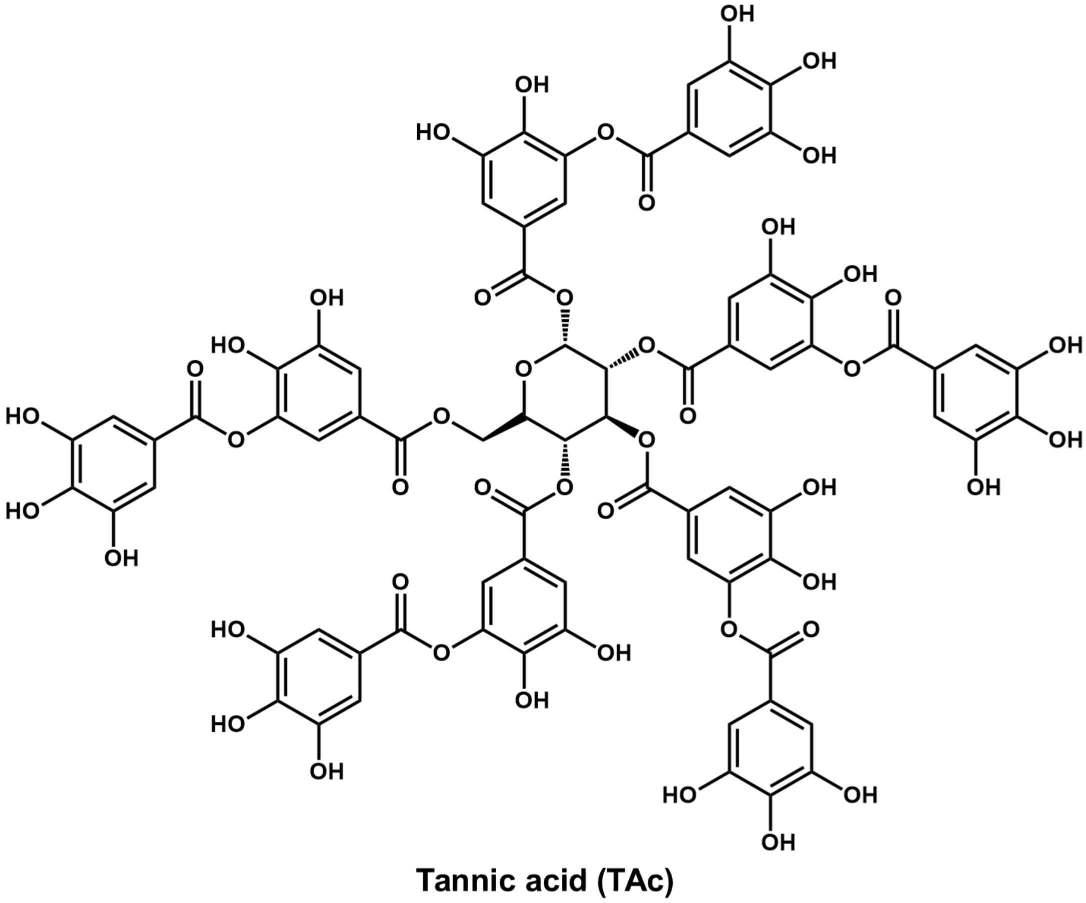

Figure S1. Molecular structure of tannic acid (TAc).

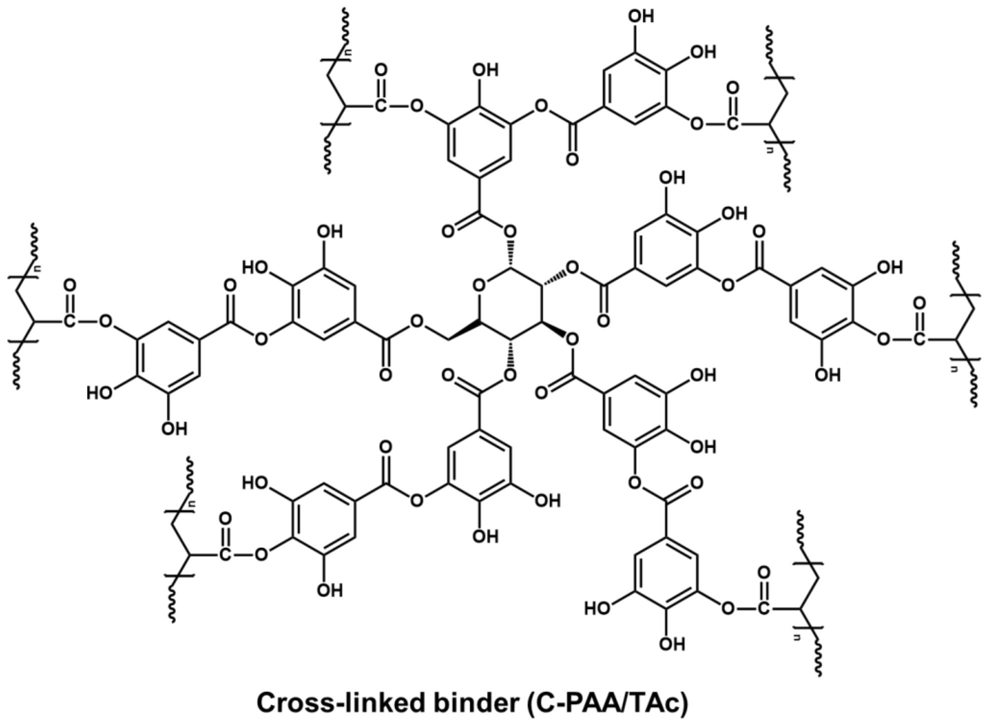

Figure S2. Cross-linked binder (C-PAA/TAc) formed by condensation between PAA and TAc.

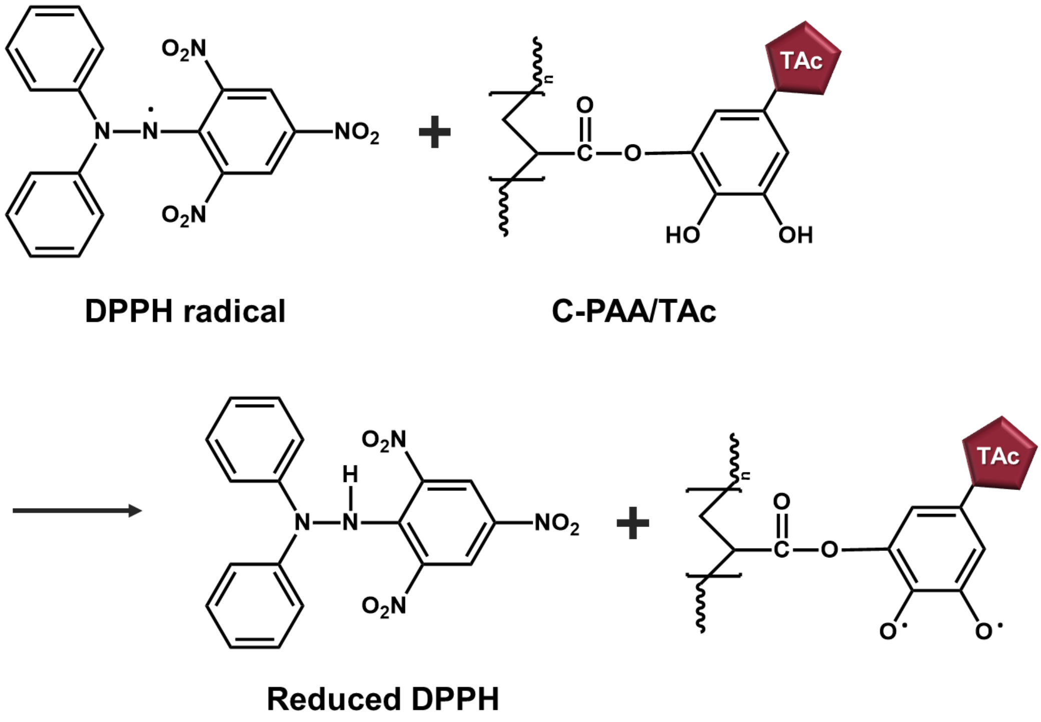

Figure S3. Reaction of antioxidant and DPPH free radical.

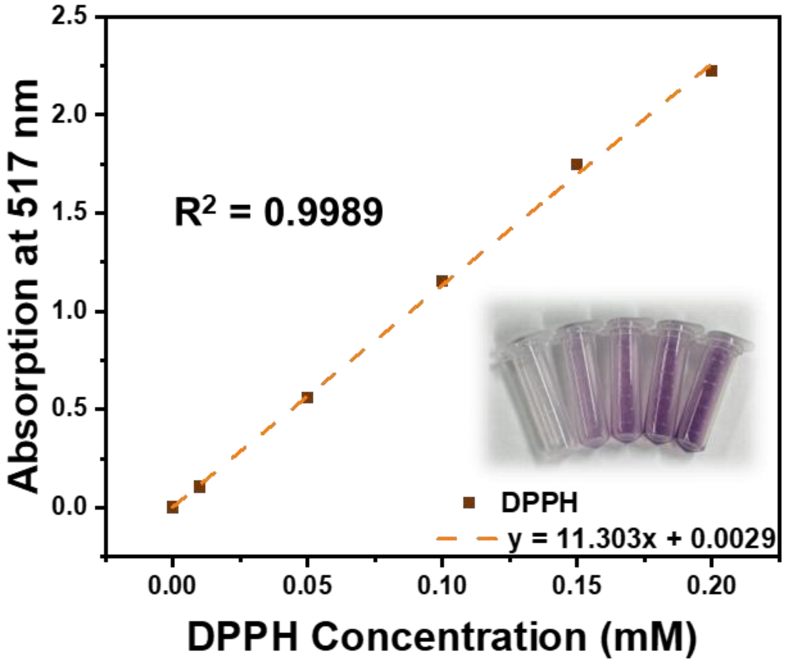

Figure S4. Optimization of DPPH assay: Color response as a function of the DPPH concentration.

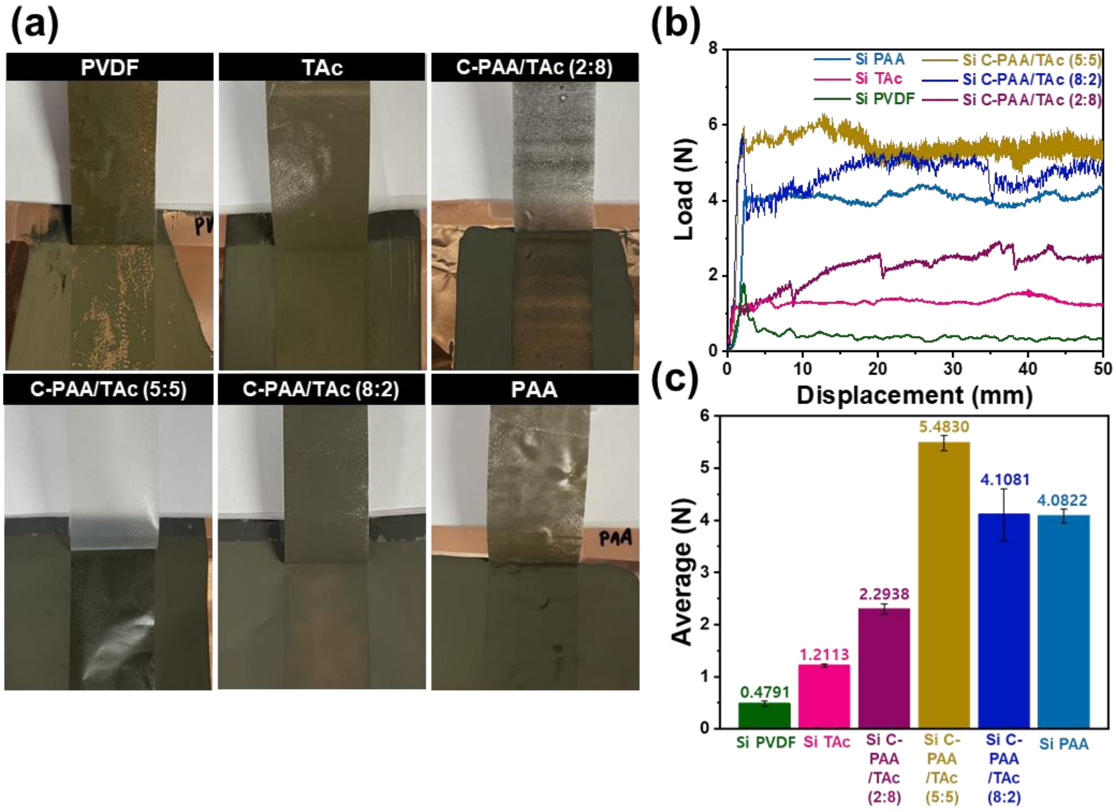

Figure S5. (a) Optical images of the tapes peeled from the Si PVDF, Si TAc, Si C-PAA/TAc, and Si PAA electrodes; (b) 180° peel-off test results of Si PVDF, Si TAc, Si C-PAA/TAc, and Si PAA electrodes EIS data of Si PAA, Si TAc and Si C-PAA/TAc; (c) the average peeling forces.

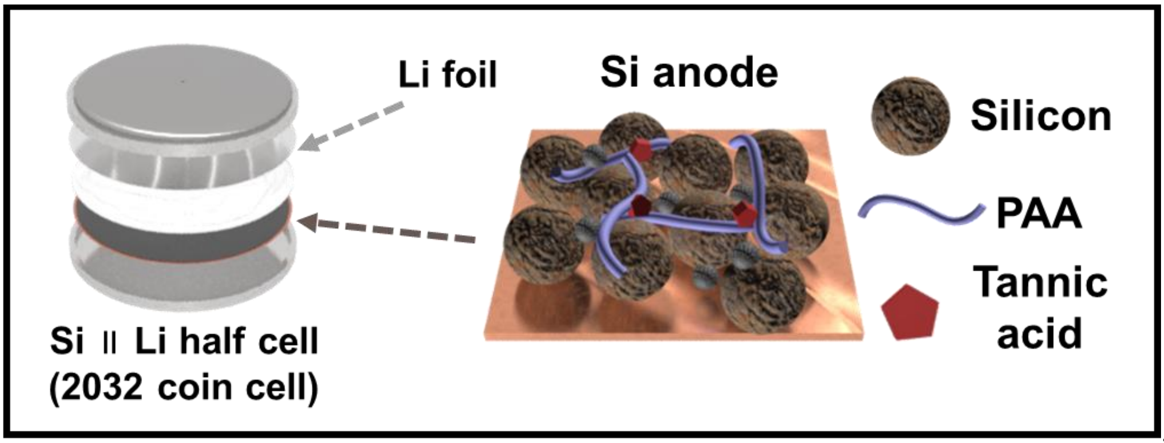

Figure S6. Schematic assembly illustration of Si ॥ Li half cell configuration.

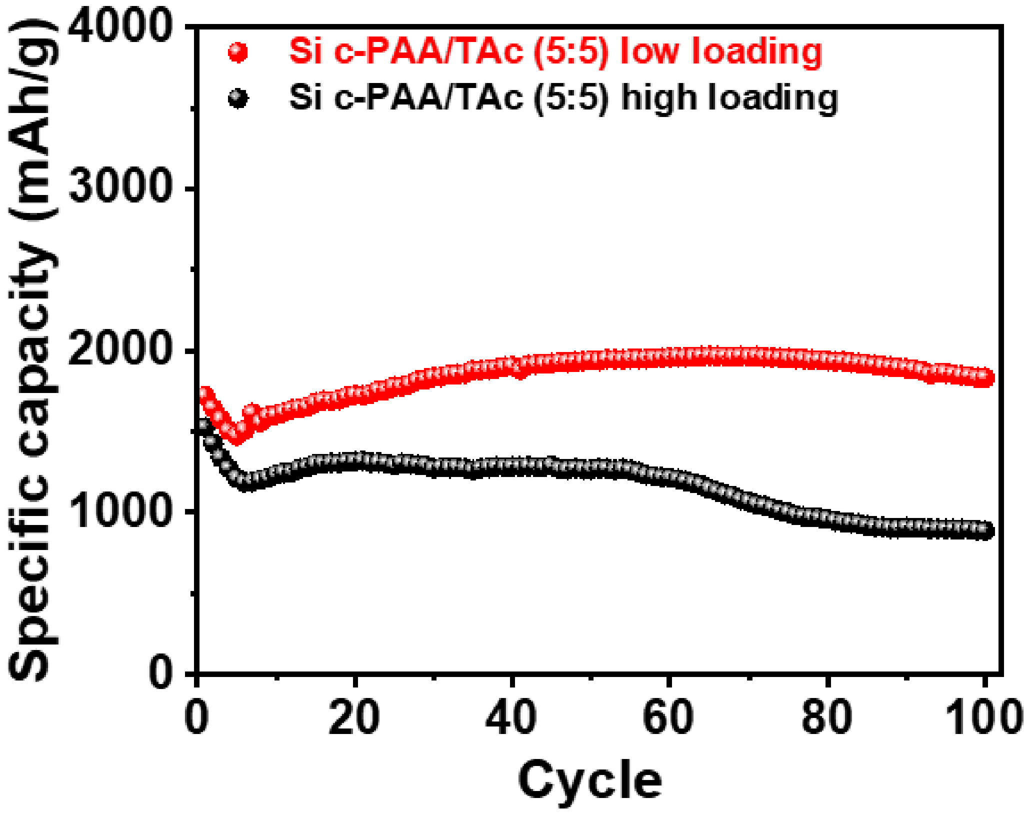

Figure S7. Cycling performance of Si C-PAA/TAc low (~ 0.35 mg cm^{-2}) and high (~1.1 mg cm^{-2}) loading at 0.5 C-rate.

Table S1. Electrochemical performances of Si PAA, Si TAc and Si C-PAA/TAc.

	1st discharge capacity (mA h g^{-1})	1st charge capacity (mA h g^{-1})	1st cycle efficiency (%)	100th cycle capacity (mA h g^{-1})	Capacity retention (100th cycle)
Si PAA	2583	2126	82.30	1072	67.61
Si TAc	2205	1617	73.33	1112	61.74
Si C-PAA/Tac (5:5)	2692	2195	81.53	1833	106.5

Si C-PAA/Tac (8:2)	2706	2235	82.59	1545	90.35
Si C-PAA/Tac (2:8)	2595	2061	79.42	1193	69.36

Table S2. EIS data of Si PAA, Si TAc and Si C-PAA/TAc after 100th cycle.

	**R_S at 100th cycle**	**R_{SEI} at 100th cycle**	**R_{CT} at 100th cycle**
Si PAA	8.57	11.23	144.33
Si TAc	3.01	7.17	85.76
Si C-PAA/Tac (5:5)	2.84	5.98	85.10
Si C-PAA/Tac (8:2)	6.11	5.99	70.27
Si C-PAA/Tac (2:8)	4.82	7.69	116.27
